# Hyperprogressive Disease in Cancers Treated With Immune Checkpoint Inhibitors

**DOI:** 10.3389/fphar.2021.678409

**Published:** 2021-07-05

**Authors:** Pan Shen, Liang Han, Xin Ba, Kai Qin, Shenghao Tu

**Affiliations:** Department of Integrated Traditional Chinese and Western Medicine, Tongji Hospital, Tongji Medical College of Huazhong University of Science and Technology, Wuhan, China

**Keywords:** immune checkpoint inhibitors, hyperprogressive disease, immunotherapy, predictive biomarker, pseudoprogression

## Abstract

Immunotherapy, which takes advantage of the immune system to eliminate cancer cells, has been widely studied and applied in oncology. Immune checkpoint inhibitors (ICIs) prevent the immune system from being turned off before cancer cells are eliminated. They have proven to be among the most promising and effective immunotherapies, with significant survival benefits and durable responses in diverse tumor types. However, an increasing number of retrospective studies have found that some patients treated with ICIs experience unusual responses, including accelerated proliferation of tumor cells and rapid progression of the disease, with poor outcomes. Such unexpected adverse events are termed hyperprogressive disease (HPD), and their occurrence suggests that ICIs are detrimental to a subset of cancer patients. HPD is common, with an incidence ranging between 4 and 29% in several cancer types. However, the mechanisms of HPD remain poorly understood, and no clinical predictive factors of HPD have been identified. In this review, we summarize current findings, including retrospective studies and case reports, and focus on several key issues including the defining characteristics, predictive biomarkers, potential mechanisms of HPD, and strategies for avoiding HPD after ICI treatment.

## Introduction

Cancer is a prevalent disease that threatens human health worldwide (Bray et al., 2018). The immune escape of cancer cells is a significant challenge in tumor treatment. In-depth studies of the molecular interactions between tumors and immune cells have provided new prospects for tumor treatments. In recent years, nivolumab, pembrolizumab, durvalumab, and atezolizumab have been approved for the treatment of non-small-cell lung cancer (NSCLC) and head and neck squamous cell carcinoma (HNSCC) (Hodi et al., 2010; Robert et al., 2015; Herbst et al., 2016; Di Pilato et al., 2019). Immune checkpoint blockade reestablishes the anti-tumor response and prevents tumor cells from evading immune surveillance by targeting specific molecules such as programmed death receptor 1 (PD-1) or its ligand (PD-L1) and cytotoxic T lymphocyte antigen 4 (CTLA-4) (Ribas and Wolchok, 2018). Immune checkpoint inhibitors (ICIs) have been referred to as a breakthrough therapy in some cancer types, and their application has led to a new era in immunotherapy. A total of ten anti-PD-1/PD-L1 antibodies, including pembrolizumab, treprizumab, nivolumab, atezolizumab, sintilimab, durvalumab, carrelizumab, tilelizumab, avelumab, and cemiplimab, as well as one anti-CLTA-4 antibody, ipilimumab has been approved for the clinical application in United States and China. These agents are different from conventional cytotoxic therapies and molecular targeted drugs in many respects; their effects include delayed tumor regression, long-term survival benefits, and pseudoprogression (Soria et al., 2018). However, some patients experience rapid disease progression and deterioration. This particular type of accelerated tumor progression has been described as hyperprogressive disease (HPD) (Champiat et al., 2017; Kato et al., 2017; Saâda-Bouzid et al., 2017; Weiss et al., 2017; Faure et al., 2018; Ferrara et al., 2018; Matos et al., 2018; Boland et al., 2019; Costantini et al., 2019; Ji et al., 2019; Kanjanapan et al., 2019; Kim C. G. et al., 2019; Lo Russo et al., 2019; Lu et al., 2019; Sasaki et al., 2019; Wong et al., 2019; Arasanz et al., 2020; Petrioli et al., 2020; Refae et al., 2020; Ruiz-Patiño et al., 2020) (Table 1). Thus, ICI immunotherapy may be not only ineffective in some patients but also harmful. Several doctors and investigators have studied this phenomenon in practice and found that a subset of advanced cancer patients treated with ICIs are more likely to experience a severe decline in their quality of life and poor prognosis. According to previously published studies, the incidence of HPD ranges from about 4 to 29%. In addition, only 15–40% of patients benefit from ICIs (Borghaei et al., 2015), and some experience relatively long-lasting adverse responses. The emergence of HPD poses a new challenge to the current approach used to evaluate the efficacy of ICIs. Various diagnostic tools and criteria are used to assess disease progression, including the immune response evaluation criteria in solid tumors (iRECIST) (Seymour et al., 2017), immune-related response evaluation criteria in solid tumors (irRECIST) (Nishino et al., 2013), and immune-related response criteria (irRC) (Wolchok et al., 2009).

**TABLE 1:** Studies on HPD after ICI treatment.

References	Tumor types	Therapy of ICIs	HPD criteria	Previous therapies	HPD incidence	HPD predictors/factors	Study design
Champiat et al. (2017)	Multiple cancer types	PD-1/PD-L1 inhibitors	1. RECIST 1.1 progression	Chemotherapy/radiotherapy/targeted therapy/immunotherapy	9% (12/131)	Older age (≥65) (*p* = 0.007)	Phase I trials
			2. TGR ≥ 2-fold increase				
Kato et al. (2017)	Multiple cancer types (Melanoma, 33%. NSCLC, 25%)	PD-1/PD-L1/CTLA-4 inhibitors, other investigational agents	1. TTF < 2 months	Chemotherapy/radiotherapy/targeted therapy/immunotherapy<	3.9% (6/155)	1. EGFR alteration (*p* = 0.005)	Genomic Analysis
			2. Progression pace > 2x			2. MDM2/MDM4 amplification (*p* = 0.007)	
			3. Tumor burden increase >50% compared with baseline			—	
Saâda-Bouzid et al. (2017)	R/M HNSCC	PD-1/PD-L1 inhibitors	TGK_R_ ≥ 2	—	29.4% (10/34)	1. Locoregional recurrence (*p* = 0.008)	Retrospective
						2. Presence of cervical nodes at diagnosis	
Weiss et al. (2017)	Multiple cancer types (Melanoma, 22%)	PD-1/PD-L1/CTLA-4 inhibitors	—	Chemotherapy/targeted therapy/radiotherapy/immunotherapy	10.7% (6/56)	Chromosomal instability quantification in plasma of cell-free DNA	Retrospective
Faure et al. (2018)	Anorectal malignant melanoma	PD-1 inhibitors	—	Chemotherapy	Case report	A role of monocytes	Retrospective
Ferrara et al. (2018)	NSCLC	PD-1/PD-L1 inhibitors	1. RECIST 1.1 progression	Chemotherapy/radiotherapy	13.8% (56/406)	Metastatic sites >2 (*p* = 0.006)	Retrospective
			2. Disease progression with change in TGR >50%				
Matos et al. (2018)	Multiple cancer types	PD-1/PD-L1/CTLA-4 inhibitors	TTF <2 months and increase in measurable lesions of 10 mm plus increase of ≥40% in target tumor burden compared with baseline or increase ≥20% in target tumor burden plus multiple new lesions	—	15.4% (33/214)	—	Phase I trials
Boland et al. (2019)	Epithelial ovarian cancer	PD-1/PD-L1/CTLA-4/LAG3 inhibitors	—	—	33.7% (30/89)	1. Neutrophil-to-lymphocyte ratio (NLR) >4 (*p* = 0.017)	Retrospective
						2. Liver parenchymal metastases (*p* = 0.001)	
Costantini et al. (2019)	Advanced NSCLC	PD-1 inhibitors	Patients receiving less than three injections of nivolumab	Radiotherapy	19.5% (57/292)	PS ≥ 2, Shorter duration of treatment before nivolumab (*p* < 0.0001)	Retrospective
Ji et al. (2019)	Malignant tumors of digestive system	PD-1/PD-L1/CTLA-4 inhibitors	TGK_R_ ≥ 2	—	20% (5/25)	—	Retrospective
Kanjanapan et al. (2019)	Multiple cancer types	PD-1/PD-L1/CTLA-4 inhibitors	1. RECIST 1.1 progression	—	7% (12/182)	Female gender	Phase I trials
			2. TGR ≥ twofold increase				
Lo Russo et al. (2019)	NSCLC	PD-1/PD-L1/CTLA-4 inhibitors	RECIST 1.1 progression and at least 3 of: 1. TTF <2 months	—	25.7% (39/152)	Clustered macrophages with epithelioid morphology and colocalization of CD163, PD-L1, and CD33 markers	Retrospective
			2. ≥50% increase of sum of target lesions major diameters				
			3. At least two new lesions in an organ already involved				
			4. ECOG PS ≥ 2				
Sasaki et al. (2019)	Advanced gastric cancer	PD-1 inhibitors	TGK ≥ 2	Chemotherapy/radiotherapy	21.0% (13/62)	Neutrophil count increased (*p* = 0.002)	Retrospective
						Increased CRP levels (*p* = 0.006)	
Wong et al. (2019)	hepatocellular carcinoma (HCC)	PD-1/CTLA-4 inhibitors	TGR ≥ 2-fold increase	Chemotherapy/radiotherapy	—	Previous radiotherapy treatment	Case series
Petrioli et al. (2020)	Urothelial transitional cancer (UTC), lung adenocarcinoma, and HCC	Nivolumab	Two-fold or greater increase in the TGR	Chemotherapy	6.4% (3/47)	1. Higher LDH serum levels	Case series
						2. NLR >3	
Kim C. G. et al. (2019)	NSCLC	PD-1 or PD-L1 inhibitors	Post-TGR/pre-TGR > 2 or post-TGK/pre-TGK > 2	Chemotherapy/targeted therapy	19.0% (45/237)	TIGIT + PD1+ CD8^+^ T cells increased	Retrospective
Lu et al. (2019)	Gastric cancer, esophageal cancer, colorectal cancer	PD-1 or PD-L1 inhibitors monotherapy or combined with CTLA-4 inhibitor	Post-TGK/pre-TGK ≥ 2	Chemotherapy/radiotherapy	8.9% (5/56)	Lower serum MCP-1 and leukocyte inhibition factor levels	Prospective
Arasanz et al. (2020)	NSCLC	PD-1 and/or PD-L1 inhibitors	TGR ≥ 2-fold increase	Chemotherapy/radiotherapy	17.9% (10/56)	Higher levels of CD28^−^ CD4^+^ T lymphocytes	Prospective
Refae et al. (2020)	NSCLC, head and neck cancer, melanoma, RCC, and others	PD-1 or PD-L1 inhibitors alone	Post-TGR/pre-TGR > 2	—	13.8% (11/80)	1. Older age (≥70)<*p* = 0.0025)	Retrospective
						2. Variations with rs2282055 (PD-L1) and rs1870377 (VEGFR2)	
Ruiz-Patiño et al. (2020)	NSCLC	PD-1 or PD-L1 inhibitor monotherapy or combined with CTLA-4 inhibitor or chemotherapy	Two-fold increase in TGR	—	19.8% (44/222)	—	Retrospective

Currently, there is no consensual definition of HPD. Previous studies have explored its occurrence and biomarkers and the molecular mechanisms underlying the role of ICIs in HPD. This review aims to discuss unexplored questions and mechanisms relevant to HPD and summarizes the current data on diagnostic tools and potential biomarkers for HPD.

## Evidence of Hyperprogressive Disease and Controversy

In 2016, Champiat et al. first reported HPD with an incidence of 9% (12/131) in a phase 1 trial of anti-PD-1/PD-L1 inhibitors (Champiat et al., 2017). In this study, HPD was not associated with an increased tumor load at baseline or specific types of tumors but was found to occur more frequently in patients over 65 years of age. A retrospective analysis of 34 patients with HNSCC by Saada-Bouzid et al. reported a maximum incidence of 29% (Saâda-Bouzid et al., 2017). In the clinical trials CheckMate 057 (28), CheckMate 227 (Hellmann et al., 2018), and CheckMate 141 (Ferris et al., 2016) that compared ICIs with standard chemotherapy, the crossing of overall and disease-free survival curves was proposed as evidence for HPD. In recent years, a study by Lahmar et al. found that 10% of NSCLC patients developed HPD (Lahmar et al., 2016). In a cohort of 242 NSCLC patients, the incidence of HPD was 16% (Ferrara et al., 2017). Although the clinical diagnostic criteria for HPD varied between studies, tumor growth was compared before and after the initiation of immunotherapy within a short time.

As HPD clinical studies are mostly retrospective, whether HPD is an independent pattern of post-treatment responses to ICIs remains controversial. The main dispute is over whether HPD is a natural process of tumors or an accelerated growth process that occurs after ICI treatment (Pearson and Sweis, 2019). In most trials, there is no reference data on the tumor growth rate (TGR) before initiation of ICIs. Moreover, the crossing of the two survival curves is considered to occur because chemotherapy works more efficiently than ICIs, rather than because of HPD itself. Gandara et al. reported a similar proportion of fast-progressing patients in a cohort of 850 NSCLC participants treated with docetaxel or atezolizumab; they suggested that HPD may be caused by a poor prognosis, not by the immunotherapy (Gandara et al., 2018). Moreover, despite accumulating data on HPD, its definition is not universal. Future studies should assess the optimal HPD criteria to identify patients who cannot benefit from ICI treatments and those who are most likely to benefit from these costly and potentially toxic treatments. It is important to collect imaging treatment and tumor growth kinetic data before ICI therapy and to distinguish HPD from an inherently aggressive disease or pseudoprogression.

## Definition and Diagnosis of Hyperprogressive Disease

In contrast to pseudoprogression, tumor growth is not caused by increased inflammation but by the specific action of ICIs as enhancers of tumor progression. It is essential to detect and distinguish progression, pseudoprogression, and HPD at an early stage. Currently, the assessment and diagnosis of HPD are mainly based on parameters related to pretreatment tumor kinetics and early changes after the start of immunotherapy, including the TGR, tumor growth kinetics (TGK), and time to treatment failure (TTF). HPD was first described in case reports and retrospective studies involving patients with accelerated tumor growth after ICI treatment. According to RECIST 1.1, HPD can be defined by a ≥2-fold increase in TGR after immunotherapy (Kanjanapan et al., 2019), whereas Ferrara et al. established a different criterion of the TGR increasing by 50% (Ferrara et al., 2018). The TGR is a ratio of the change in tumor size over a given time interval (Gomez-Roca et al., 2011; Ferté et al., 2014) and is a predictor of overall survival in clinical practice (Ten Berge et al., 2019). Assessment of the TGR is based on RECIST, which assesses changes in the sum of the largest diameter of target lesions at multiple time points and uses a natural logarithm for correction. The lack of an association between RECIST and changes in TGR before and after treatment suggests that RECIST provides limited information (Gomez-Roca et al., 2011). Therefore, RECIST 1.1 is not the most accurate way to evaluate the efficacy of immunotherapy. In fact, the use of RECIST 1.1 was found to underestimate the positive response rate in 160 NSCLC patients and indicated a 15% treatment benefit rate in 655 melanoma patients treated with ICIs.

The evaluation of TGK is similar to that of TGR: changes in the sum of the largest diameter of the target lesion are also measured, but no logarithm correction is performed. HPD is defined by a ≥2-fold increase in the TGK of the target lesion at the time of the first evaluation compared with before ICI treatment (Saâda-Bouzid et al., 2017). According to RECIST, HPD assessment requires tumor burden data to be obtained at earlier time points for the first TGR assessment, and two computed tomography scan evaluations are needed. However, this HPD evaluation method is limited to target lesions of the tumor and does not consider the appearance and changes of non-target lesions. In actual clinical practice, TGR data before immunotherapy cannot be obtained, and the rate cannot be distinguished by basic imaging analysis of changes in tumor size. Patients with HPD have no clinically detectable tumor growth prior to their first treatment, and the maximum tumor single diameter is unavailable; therefore TGR and TGK cannot not be assessed. TGR- and TGK-based methods cannot be used to evaluate new lesions in the assessment of tumor growth. Importantly, these methods use only radiological criteria, which may lead to misclassification of response patterns. Previous studies have used a combination of clinical and radiological criteria. A TTF of less than 2 months is also used as a surrogate indicator for evaluating HPD. Kato et al. (2017) provided an additional two criteria: an increase of more than 50% of tumor burden compared with pre-immunotherapy imaging, and a 2-fold or greater increase in progression pace. Kato et al. found that melanoma patients had longer TTF compared with patients with other tumor types; this may suggest either that patients with melanoma are less likely to develop HPD or that TTF is not an effective diagnostic marker for HPD.

Zuazo-Ibarra et al. quantified the number of circulating senescent CD4^+^ T cells (Tsens) before ICI treatment. Increased numbers of Tsens before immunotherapy indicate a response, whereas a decrease in the number of Tsens after the first treatment cycle indicates a good response. The decrease may be due to G1 phase withdrawal or tumor cell recruitment from the blood. Conversely, proliferation of Tsens suggests progression. The authors concluded that extensive validation is necessary to apply these findings in NSCLC and more widely in clinical practice (Zuazo et al., 2018). Boeri et al. (2018) identified potential prognostic value in the immune environment by analyzing microRNAs in plasma samples. In general, technologies based on liquid biopsy are expected to provide reliable methods for stratification and disease tracking for clinical applications, including future real-time applications. Combinations of several methods provide the most valuable assessments. Despite the relative inaccuracy of HPD diagnosis, the current results are still informative for further studies.

## What is Pseudoprogression?

In brain cancer, the concept of tumor pseudoprogression was first proposed in patients treated with the non-immunotherapeutic agent temozolomide. However, this phenomenon was not accompanied by real tumor progression, and the brain tumor may have grown before temozolomide treatment (Brandsma and van den Bent, 2009). Although pseudoprogression has rarely been observed in patients treated with conventional cytotoxic drugs, it occurs relatively frequently during ICI therapy. Pseudoprogression in patients treated with ICIs was first identified in a melanoma study of the anti-CTLA4 inhibitor ipilimumab (Wolchok and Saenger, 2008) and then in subsequent studies of the anti-PD-1 inhibitors pembrolizumab and nivolumab (Chiou and Burotto, 2015). Patients with pseudoprogression and HPD have completely different outcomes. Moreover, pseudoprogression can either occur within the first 12 weeks of treatment or be delayed. Given that beneficial treatments are often discontinued in initial trials because of pseudoprogression, it is essential to distinguish between pseudoprogression and HPD. Pseudoprogression is not true tumor progression but radiographic growth pathologically characterized by infiltration, edema, and necrosis of immune cells surrounding the tumor. Hodi et al. considered pseudoprogression to be an increase in the tumor burden of at least 25% with no representative progressive disease detected in subsequent assessments (Hodi et al., 2016). In addition to the inflammatory response induced by tumor immune cell invasion, delayed immune responses may also play a part in pseudoprogression, especially in patients with tumor regression after pseudoprogression. There have been several studies on the incidence of pseudoprogression when ICIs are used to treat solid tumors, although this is relatively rare. In melanoma, an unusual immune response or pseudoprogression followed by a delayed response was observed in 3.7–15.8% of patients treated with ICIs (Hodi et al., 2008; Ribas et al., 2012; Millward et al., 2013). The incidences of pseudoprogression in patients with NSCLC, urothelial carcinoma, HNSCC, and mesothelioma have been reported to be 0.6–5.8, 1.5–7.1, 1.8, and 6.9%, respectively. Although pseudoprogression mostly occurs in patients receiving single checkpoint inhibitors, it has also been observed in patients receiving dual immunotherapy. In a case report, a patient with microsatellite unstable high metastatic colorectal cancer treated with the combination of a PD-L1 antagonist and OX40 agonist showed pseudoprogression, with a 163% increase in the baseline tumor burden (Chae et al., 2017). Tumor atrophy was subsequently observed, and the patient’s condition was stable. This large increase in tumor size presents a challenge in differentiating pseudoprogression from authentic progression (Wolchok et al., 2009). Therefore, clinicians should depend on other information to accurately assess tumor status. To address this issue, new criteria, including irRC, irRECIST, and iRECIST, were applied to differentiate true progression from pseudoprogression. The patient was re-examined 4 weeks after the diagnosis of an underlying progressive disease to ensure that it was not spurious progression (Seymour et al., 2017).

Moreover, the analysis of cell-free circulating tumor DNA (ctDNA) levels might be used as an effective tool in distinguishing progressive disease, HPD, and pseudoprogression (Lipson et al., 2014; Cabel et al., 2018). It evaluates the alternation in ctDNA levels to separate cell-free DNA from plasma for liquid biopsy, thereby detecting the changes of tumor-specific copy number and genome instability number. Pseudoprogression showed as a decrease in genome instability in ctDNA, unlike HPD (Jensen et al., 2019). In a study of 125 patients with melanoma received ICI treatment (Lee et al., 2018), ctDNA profiles distinguished pseudoprogression and HPD with high sensitivity (90%) and specificity (100%).

**FIGURE 1:**
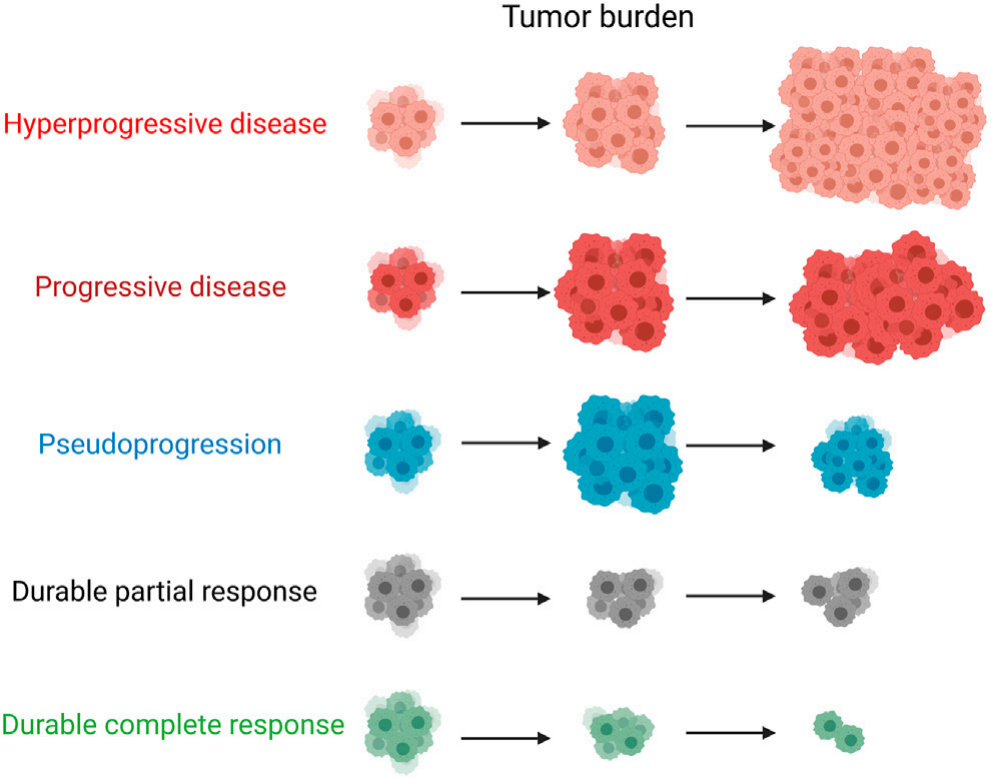
Patterns of response and progression with immune checkpoint inhibitor treatment.

It is believed that pseudoprogression has a favorable prognosis and should be identified as soon as possible to avoid delaying the disease owing to the early interruption of treatment. At the same time, it is important to be aware of the possibility of real tumor progression (Figure 1).

## Biological and Clinicopathological Factors of Hyperprogressive Disease

It is essential to identify potential predictive factors of HPD. This can enable the adverse effects caused by ICIs to be avoided and has important clinical significance in prolonging the survival period and quality of life of patients. Champiat et al. (2017) and Saâda-Bouzid et al. (2017) found no association between HPD and baseline tumor load, previous treatment line, tumor histology, type of immunotherapy, or number of metastatic sites. To date, five clinical variables—aging, female sex, higher serum lactate dehydrogenase (LDH) concentration, metastasis burden, and local recurrence of the cells in the irradiation area—have been identified as potentially associated with HPD, as have specific genomic mutations including *MDM2/MDM4* amplification and *EGFR* aberrations.

In the study reported by Champiat et al., older age appeared to be related to the occurrence of HPD. Patients with HPD were significantly older than those without HPD, with 19% of patients over 65 showing HPD compared with 5% of patients younger than 64. These results indicate that older patients benefit less from ICIs than younger people. T cell immunity declines with age because of changes in T cell number, diversity, phenotype, and function (Fulop et al., 2017). In addition, T cell signaling through T cell receptors has been found to decrease with age (Solana et al., 2012; Goronzy and Weyand, 2013). However, this was not observed in two other studies. Therefore, the specific relationship between HPD and aging is not completely clear.

Saada-Bouzid et al. found that HPD was related to radiotherapy. In a previous anti-PD-1/PD-L1 study, 50% of patients with regional recurrence had HPD, whereas only 6.25% of patients without regional recurrence had HPD. Almost all cases of HPD occurred in patients with recurrence in the irradiated area, but the underlying mechanism of this phenomenon is unclear. Ferrara et al. found that HPD was more frequent in NSCLC patients with more than two metastatic sites (Weiss et al., 2017). Kanjanapan et al. found that the incidence of HPD in women was significantly higher than that in men; this was the only study in which HPD was shown to be associated with gender (Kanjanapan et al., 2019). Several studies found that regional recurrence in HNSCC was associated with a higher risk of HPD. Sasaki et al. reported that liver metastases, an early increase in neutrophil counts and C reactive protein levels, and a large sum of target lesion diameters at baseline were associated with HPD risk (Sasaki et al., 2019). The local recurrence of HNSCC and metastasis of most other types of cancer are associated with poor prognosis, because in these cases the tumor has successfully acquired the characteristics necessary for survival at the primary site and metastasis to a distant location. Therefore, it is difficult to distinguish the relevant prognostic factors and predictors.

Kato et al. analyzed genomic mutations (155 patients) as potential genomic markers associated with immunotherapy and HPD using next-generation sequencing (Kato et al., 2017). A favorable clinical outcome (TTF ≥ 2 months) was observed in patients with several genetic alternations, including mutations in *TERT* [odds ratio (OR): 0.42; *p* = 0.07], *PTEN* (OR: 0.28; *p* = 0.10), *NF1* (OR: 0.15; *p* = 0.07), and *NOTCH1* (OR: <0.19; *p* = 0.02). Conversely, *EGFR* (OR: 10.2; *p* = 0.002), *MDM2/4* (OR: > 11.9; *p* = 0.001), and *DNMT3A* (OR, 9.33; *p* = 0.03) alterations were related to worse outcomes (TTF < 2 months). Six patients with alterations exhibited an HPD phenotype, and all had a TTF < 2 months, whereas 20% of patients with *EGFR* alterations exhibited an HPD phenotype. Of five patients with *DNMT3A* alterations, only one was radiologically evaluable and did not have an HPD phenotype. In a study by Singavi et al., the incidence of HPD in patients with *EGFR* amplification was 50% (Singavi et al., 2017). *EGFR* mutations are likely to be associated with the upregulation of PD-1/PD-L1, which can activate immune escape. If the results obtained from larger cohorts are consistent with the current findings, *MDM2/4* and *EGFR* alternations could potentially be used as reliable HPD predictive biomarkers.

Lo Russo et al. (2019) did not find significant differences in tumor-infiltrating T cells of the following types: CD4+/CD8+ lymphocytes, regulatory T cells (Tregs), peritumoral and stromal myeloperoxidase myeloid cells, and PD-1+ and PD-L1+ immune cells. However, HPD was associated with the density of myeloperoxidase myeloid cells within the tumor and inversely correlated with PD-L1 expression in tumor cells. Some investigators focused on CD8^+^ T cells in the peripheral blood to find potential predictors. Studies have found that number of effector CD8^+^ T cells (CCR7−CD45RA−) decreased (Jenkins et al., 2018), whereas number of exhausted tumor-reactive CD8^+^ T cells (TIGIT + PD-1+) increased in HPD patients with NSCLC (Huang A. C. et al., 2017). The results suggest that the exhaustion of CD8^+^ T cells is one of the potential mechanisms that triggers the acceleration of tumor growth with ICIs treatment. The severity of T cell exhaustion can be predictors for HPD. In addition, HPD patients also showed a significant upregulation in the number of CD28^−^CD4^+^ cells after immunotherapy (Arasanz et al., 2020). The number of CD62LlowCD4+ effector Th1 cells was significantly higher in the peripheral blood of patients with NSCLC before treatment with PD-1 inhibitor, while a decrease in CD62LlowCD4+ T cells was related with acquired resistance (Kagamu et al., 2020), indicating that CD4^+^ T cell immunity could be a powerful predictor in HPD.

Weiss et al. and Jensen et al. performed genome-wide sequencing of plasma/serum-derived cell-free DNA and found that genomic copy number instability (CNI) could help to determine HPD (Weiss et al., 2017; Jensen et al., 2019); however, their study included a small number of patients. Further prospective research should be conducted to determine whether CNI could be a novel marker for HPD. In an exploratory study of HPD, NSCLC patients with an increased proportion of depleted T cell subtypes and a decreased proportion of effector T cells showed a higher incidence of HPD and worse prognosis. These results suggest that T cell subtypes help predict the occurrence of HPD (Ferrara et al., 2018). Lo Russo et al. (2019) compared patient responses and histopathological and molecular expression patterns in 35 patients with advanced NSCLC after immunotherapy. The results showed that HPD was associated with the number of myeloperoxidase-positive myeloid cells in the tumor and inversely associated with PD-L1 expression in tumor cells. In patients with HPD, the presence of CD163 + CD33+PD-L1+ macrophages with an epithelioid morphology was identified more frequently than in patients without HPD, suggesting that these macrophages are important in the HPD process. In a study, infiltration of epithelioid-shaped macrophages in tumor tissues was observed in 104 NSCLC patients who experienced HPD (Lo Russo et al., 2019). Tunali et al. established a clinical prediction model by combining extensive clinical-pathological, laboratory, and imaging data to predict the occurrence of HPD in NSCLC patients (Tunali et al., 2019).

Owing to the limited size and scope of previous cohort studies, we cannot draw reliable conclusions to accurately assess risks and benefits in specific patient groups. Furthermore, the current research hypothesis needs to be further verified. Tumor types, stages, and other covariates should be controlled, especially in studies that investigate covariates and their associations with patient response or resistance to immunotherapy.

## The Main Hypotheses Regarding the Mechanisms Underlying Hyperprogressive Disease

**FIGURE 2:**
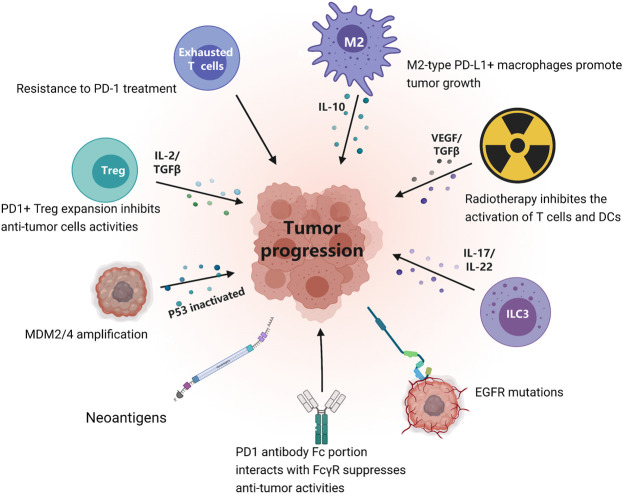
Potential mechanisms of HPD under immune checkpoint inhibitor treatment.

At present, the molecular mechanisms of HPD remain elusive. Several studies have explored multiple hypotheses or mechanisms of HPD from different perspectives (Figure 2; Table 2). These mechanisms may act independently or be complementary. It is of great significance to clarify the molecular mechanism of HPD. HPD can be caused by a variety of factors, including the characteristics of tumor cells, the status of the patient’s immune system, and the patient’s current or previous treatment history.

**TABLE 2:** Potential mechanisms of HPD in different types of tumors.

Mechanisms	Tumor types
Upregulation of PD-1 + Tregs	Multiple cancer types
T Cell exhaustion	Metastatic ovarian cancer, Head and neck cancer
Increased Tsens	NSCLC
*MDM2/4* amplification and *EGFR* mutations	Multiple cancer types
The effects of Fc fragment of macrophages and ICIs	Multiple cancer types
Imbalance of immunosuppressive cytokines and factors	Gastric cancer, Ovarian cancer, NSCLC
Increase in ILC3	Breast cancer, Colon cancer
The effects of neoantigens	Multiple cancer types
Circulating LDH levels	UTC, Lung cancer, HCC
Radiotherapy	HCC

### Upregulation of PD-1+ Tregs

In the context of infection or cancer, contra-suppression or immune compensation is a self-stabilizing mechanism. Immunosuppressive factors such as Tregs can maintain anti-infection or anti-tumor immunity and reduce the adverse effects of ICIs (Lehner, 2008; Barnaba and Schinzari, 2013). Studies have confirmed that inhibiting the expression of PD-1 in mice upregulates other immune checkpoints, including CTLA-4, LAG3, and TIGIT, or induces the activation and proliferation of Tregs, resulting in impaired immune killing ability (Ellestad et al., 2014; Huang R. Y. et al., 2017). FOXP3 is a classical marker of Tregs, which participate in inducing immune tolerance. Nair et al. found that pembrolizumab inhibited peripheral Treg differentiation and reduced FOXP3 expression through the mTOR pathway *in vitro* (Sasidharan Nair et al., 2020). A previous study analyzed gastric cancer tumor tissues before and after ICI treatment and found increased numbers of infiltrating effector Tregs (Ki67 + Tregs) in tissues from HPD patients, suggesting that these Tregs may be activated by ICI therapy (Kamada et al., 2019). Compared with patients without HPD, the number of tumor-infiltrating Ki67 + Tregs was significantly increased in HPD patients. In an *in vitro* gastric cancer study, it was observed that when PD-1 was knocked out or the binding of PD-1 to its ligand was inhibited in Tregs, Tregs could proliferate and promote anti-tumor immune cells and tumor suppression. In mouse models, the results also support a similar effect of Tregs in HPD. From the above studies, PD-1 may mediate the occurrence of HPD, leading to the inhibition of anti-tumor immunity.

### T Cell Exhaustion

T cell dysfunction is defined as T cell exhaustion, in which the ability to recognize and eliminate antigens is weakened, and inhibitory receptors including PD-1, T cell immunoglobulin and mucin domain-containing protein 3 (TIM3), TIGIT, and LAG3 are upregulated. In mouse models and progressive cases, TIM-3 has been found to be upregulated (Koyama et al., 2016). In addition, the proportion of TIM-3+ cells was proportional to the duration of ICI treatment. No increase in TIM-3+ cells was detected in control cases. Therefore, overexpression of TIM-3 is likely to be a key mechanism of resistance to PD-1 treatment (Shayan et al., 2017). After failure of anti-PD-1 treatment, TIM-3 antibodies can provide a survival benefit. Similarly, CTLA-4 and LAG3 on cytotoxic CD8^+^ T cells were increased as a result of PD-1 blockade in a model of ovarian cancer (Huang R. Y. et al., 2017). With expression of the above compensatory receptors, CD8^+^ T cells show serious dysfunction in cytokine production, proliferation, and migration. In a mouse virus infection model, Odorizzi et al. found that cytotoxic CD8^+^ T cells were depleted, with large numbers of inhibitory receptors such as LAG3 and TIGIT on their cell surface (Odorizzi et al., 2015).

### Increased Tsens

In a study of NSCLC patients with low baseline numbers of senescent Tsens treated with anti-PD-1/PD-L1 agents, those with Tsens elevated by 12.4% also had HPD. Conversely, patients who showed a 14.4% decrease in Tsens experienced tumor regression (Zuazo-Ibarra et al., 2018). The results indicate that Tsens numbers in patients prior to immunotherapies could predict the risk of HPD. Tsens baseline numbers may represent a pre-existing large pool of antigen-specific central and effector T cells with potential anti-tumor capacities. The decreases in Tsens following antibody administration may indicate the mobilization of Tsens from peripheral blood to secondary lymphoid organs/tumor sites.

### *MDM2/4* amplification and *EGFR* Mutations

Studies involving multiple tumor types have shown that oncogenic activation, such as that resulting from *MDM2/4* amplification, is associated with the occurrence of HPD (Ferté et al., 2014). In malignant tumors, a study using second-generation sequencing found that the amplification rate of *MDM2/4* was about 3.9% (6/155), and that the TTF of all patients with *MDM2/4* amplification receiving immunotherapy was less than 2 months (Champiat et al., 2018). MDM2/4 blocks the p53 trans-activation domain and promotes proteasomal ubiquitin-dependent degradation of p53 (Wang Q. et al., 2018). IFN-γ can increase the expression of MDM2/4 and further inhibit p53 activity (Zhou et al., 2009; Zhao et al., 2014). However, loss of p53 activity is an essential driver of oncogenesis. ICIs can increase the production of IFN-γ at the tumor site, and it has been speculated that the MDM2/4-IFN-γ/p53 axis may mediate the occurrence of HPD (Peng et al., 2012). In about 50% of tumors, p53 is mutated or lost and inactivated. The other 50% retain wild-type p53, the normal regulation of which is disrupted by overexpression of MDM2 (Momand et al., 1998). Anti-PD-1/PD-L1 agents can activate JAK/STAT signaling, resulting in increased expression of interferon regulator 8 (IRF-8) (Schindler et al., 2007; Waight et al., 2013). IRF-8 can bind to the *MDM2/4* promoter and promote the expression of MDM2/4, but the cascade may not have a significant impact when MDM2/4 is not amplified (Zhao et al., 2014). The amplification rate of MDM2 in malignant lung, skin, and bladder tumors is lower, whereas that in sarcoma is higher, consistent with the primary resistance of sarcoma to ICIs [59]. If MDM2 amplification does cause ICI resistance or HPD, the combination of ICIs and MDM2 inhibitors may be a promising strategy to overcome resistance and HPD. In addition to MDM2/4, the activation of other oncogenic signals may also contribute to HPD after ICI therapy. Xiong et al. found increased activity of IGF-1, ERK/MAPK, and PI3K/AKT in patients with HPD after ICI therapy, whereas tumor suppressor genes including *TSC2* and *VHL* were not expressed (Xiong et al., 2018). The activation of EGFR is usually accompanied by upregulation of PD-1/PD-L1 or CTLA-4 to promote tumor immune escape (Akbay et al., 2013). The objective response rate of anti-PD-1 treatment in patients with *EGFR* mutations is relatively low at about 3.6% (Gainor et al., 2016). This may be related to the activation of EGFR, which can promote the stability of PD-L1 and prevent it from being easily degraded (Li et al., 2016). However, the mechanisms linking *EGFR* mutations with HPD remain unclear.

### The Effects of Fc Fragment of Macrophages and Immune Checkpoint Inhibitors

Immune microenvironments are closely related to HPD. NSCLC tumor tissues from patients (patient-derived xenografts, PDX) were transplanted into athymic nude or severe combined immunodeficient (SCID) mice. Nivolumab or nivolumab F (ab)2 fragments were administered to mice at the nodule. Nivolumab promoted tumor growth, whereas no substantial tumor growth or HPD was observed in mice treated with the F (ab)2 fragment of nivolumab (that is, lacking the Fc fragment) (Lo Russo et al., 2019). Further studies found that M2 macrophage aggregation only occurred in lesions after nivolumab treatment but not after the injection of nivolumab F (ab)2 fragments. In another study, compared with PDX SCID mice with wild-type EGFR, TGR and tumor cell dissemination were significantly increased in PDX SCID mice with mutated versions of EGFR after nivolumab therapy (Lo Russo et al., 2019). Similarly, when mice with EGFR-mutated PDXs were treated with nivolumab F (ab)2 fragments, there was no significant evidence of HPD or tumor dissemination. It has been suggested that the occurrence of HPD is related to the interaction between macrophages and the Fc portion of nivolumab. It is also important to consider the interaction between the different Fc regions used in various PD-1 antibodies and the distinct Fc receptor variants. In a study of malignant melanoma (Dahan et al., 2015), knocking out the Fcγ receptor (FcγR) in mice enhanced the anti-tumor effect of the anti-PD-1 antibody, directly showing a correlation between FcγR and ICIs. Lo Russo et al. suggested that FcγR IIb has a detrimental effect on anti-PD-1 immunotherapy efficacy in humans, resulting in HPD (Lo Russo et al., 2019). Zhang et al. designed two anti-PD-1 monoclonal antibodies with the same specificity but different Fc sequences; they found that FcγR I induced immune tolerance by regulating inflammatory cytokines and played a part in the production of M2 macrophages to promote tumor growth (Zhang et al., 2018). This could be addressed by destabilizing the FcR interaction and modifying the current immunotherapy strategy.

### Imbalance of Immunosuppressive Cytokines and Factors

Tumor-derived exosomes have been shown to induce PD1+ macrophages to produce IL-10 and inhibit the function of CD8+T cells (Wang F. et al., 2018). In another study, Lamichhane et al. found that inhibiting the expression of PD-1 promoted the secretion of IL-10 by dendritic cells (DCs) and further increased the expression of PD-L1 in DCs (Lamichhane et al., 2017). The analysis of tumor tissues from 104 NSCLC patients with HPD revealed a large number of M2-type PD-L1+ macrophages, which secrete IL-10 to mediate the occurrence of HPD through the depletion of the PD-1 antibody (Kanazu et al., 2018). Inhibition of PD-1 expression also increases the serum angiogenin two concentration, which can increase the number of M2 macrophages and promote tumor metastasis, angiogenesis, and immunosuppression (Wu et al., 2017). The immune-resistance of tumors can be enhanced by IFN-γ via elevated expression of PD-L1 in cancer cells (Teng et al., 2018). The activation of JAK1/STAT3 enables binding of IFN-γ to its receptor IFNGR1/IFNGR2. The release of IFN-γ by T cells could promote the selection pressure of cancer cells, resulting in acquired deficiency of the IFN-γ pathway and acquired resistance to ICIs through loss of sensitivity to IFN-γ. In addition, resistance to immunotherapy was observed with a loss of IFN-γ signaling in CD8^+^ T cells (Darvin et al., 2018). These results demonstrate the potential effects of IFN-γ in HPD.

### Increase in Type 3 Innate Lymphoid Cells

Xiong et al. showed that ILC3 were specifically increased in HPD tumors (Xiong et al., 2018). ILC3s can respond to cytokine stimulation without a specific antigen. It has been reported that ILC3 can produce IL-17 and IL-22, thereby promoting cancer progression (Fung et al., 2019). Irshad et al. showed that the presence of ILC3 in the tumor microenvironment was associated with a higher risk of lymph node metastasis in breast cancer. In a mouse model of colon cancer, reduction of IL-22 production by ILC3 impaired the progression of gastrointestinal cancers (Kirchberger et al., 2013). Inhibiting the expression of PD-1 in tumors leads to increased levels of IL-6 and IL-17 in the peripheral blood; because the main function of IL-6 and IL-17 is to promote neutrophil-mediated inflammation, this may weaken the anti-tumor immune response (Fielding et al., 2008; Miossec and Kolls, 2012). This abnormal inflammatory environment is likely to be related to the poor efficacy of ICIs, but its relationship with HPD is not yet clear.

### The Effects of Neoantigens

Mutations in the protein-coding regions produce truncated proteins called “neoantigens.” Neoantigens result in higher heterogenicity of cells, which facilitates immune cell targeting and eliminates tumor cells. Acquired resistance to ICIs and HPD can also be predicted using neoantigens. Tumor cells alter the expression of multiple immune suppressive factors, leading to acquired resistance against ICIs (Jenkins et al., 2018). Dysfunction of neoantigens might promote metastasis and recurrence of tumors. Screening of these neoantigens has the potential to predict therapeutic resistance as well as HPD.

### Other Mechanisms

As mentioned earlier, changes in tumor immune microenvironments caused by radiotherapy combined with ICIs may accelerate tumor growth. In a previous study (Saâda-Bouzid et al., 2017), almost all cases of HPD occurred in patients with at least regional recurrence in an irradiated area. Radiotherapy not only upregulates the expression of VEGF and promotes tumor angiogenesis and growth, it can also enhance the expression of TGF-β, thereby inhibiting the activation of T cells and DCs.

In most cases, it is common for patients to receive cytotoxic drugs before immunotherapy. However, traditional chemotherapy has been reported to reduce the anti-tumor efficacy of immunotherapy. This suggests that resistant clones are selected after chemotherapy treatment, and the immune system cannot detect them when ICIs are used. As there is currently no effective immune monitoring tool, it is impossible to predict the risk of HPD in patients before ICI treatment. Kim et al. reported that high LDH serum levels were significantly associated with HPD. High LDH levels represent hypoxia in the tumor and acidification of the extracellular environment (Kim J. Y. et al., 2019). High LDH levels and acidic environments may influence the function of antibodies and the conformation of antigens, thereby affecting the specificity and affinity of ICIs. Okeya et al. found that after 5 weeks of pembrolizumab treatment, a 66-year-old male smoker with advanced lung adenocarcinoma developed small-cell carcinoma combined with HPD and metastases (Okeya et al., 2019). In *in vivo* and *in vitro* studies, Kudo et al. observed the presence of both chemotherapy- and immunotherapy-activated cancer stem cells, which contributed to aggressive cell proliferation and drug resistance (Kudo-Saito et al., 2019). They proposed that HPD may depend on the proportions of cancer cells eliminated and dormant cells activated. In addition, ICIs can negatively affect the endocrine system and cause autoimmune diseases. Several studies have found that gut microbiota influence the effects of ICIs in melanoma patients. The microbiome is well known to be a key regulator of inflammation and immune responses and may have an important role in cancer therapy.

## Possible Strategies for Avoiding Hyperprogressive Disease

Owing to the poor prognosis of patients with HPD, there is an urgent need to develop strategies to reduce or eliminate harm to these patients. In clinical practice, it is necessary to pay more attention to changes to the patient’s condition and to evaluate the efficacy of treatment with ICIs. First, patients to be treated with ICIs need to be fully informed of the risks of HPD, as the incidence is high enough that some patients may refuse the treatment owing to the unfavorable consequences (Zhang et al., 2021). In addition, current tumor evaluation methods of immunotherapy do not include TGK, which is a key to the early identification of HPD. It could enable a considerable proportion of patients who meet the definition of HPD to continue to receive the same treatment, and it will not be able to block HPD in time (Champiat et al., 2018). Existing disease monitoring and evaluation systems for cancer urgently need to be changed. In salvage therapy, early identification of HPD and timely replacement of ICIs might be the only remedies to avoid risk to patients at present. However, pseudoprogression also affects the identification of HPD; therefore, apparent progression of a tumor at the first imaging after initiation of immunotherapy does not necessarily mean that the treatment must be terminated, as patients with pseudoprogression could benefit significantly from this treatment (Tazdait et al., 2018). The assessment of *MDM2/4* amplification, *EGFR* mutation and CNI score before treatment is useful for selecting patients who will probably develop HPD. However, the predictive value of these biomarkers has not been validated. In addition, it is not possibly to accurately predict HPD in patients with several of these features, including high serum LDH.

The antitumor activity of the treated NK cells (nuclear-trafficking property-genome modulator) was enhanced, and repeated administration of these NK cells attenuated PD-L1-positive tumor cells *in vivo*. The use of NK cells combined with ICIs could be an alternative option for patients with HPD (Teratake et al., 2020). In the Checkmate227 trial, patients treated with nivolumab combined with chemotherapy had a lower risk of progression than those receiving nivolumab combined with ipilimumab (Hellmann et al., 2019). Chemotherapy may help prevent patients from developing ICI resistance and HPD; this possibility deserves further research.

As the relevant studies are mostly retrospective analyses and case reports, the results are inherently limited by selection bias. Clinical trials do not usually include HPD based on radiological criteria as a pattern of response, making the evaluation of this phenomenon in large cohorts of patients difficult. Clinicians should realize that after failure of ICI treatment, subsequent treatment is likely to be ineffective (Schvartsman et al., 2017), and most HPD patients do not have the opportunity to receive subsequent treatment at all (Kim C. G. et al., 2019). Further studies are required to confirm more of the mechanisms underlying HPD, and to explore other accurate biomarkers for this paradoxical response to ICIs. Investigators should attempt to assess the HPD status patients to guide their management.

## Discussion

The application of immunotherapy has contributed to fundamental changes in the treatment of cancer. As ICIs are widely administered by physicians, better survival rates are achieved in increasing numbers of patients, especially those with solid tumors. With the rapid development of ICIs, physicians should be aware of the adverse effects and correctly assess response to tumor therapy and the challenges in patient management. Although there are still many controversies regarding HPD, it is known to occur in multiple tumor types and almost all malignant tumors, and to be related to poor prognosis. An increasing number of retrospective studies indicate that HPD is induced by ICIs. HPD is a severe adverse reaction in ICI treatment, regardless of the specific type of ICI, but the underlying mechanism and predictive indicators are not yet clear, which limits the clinical application of ICIs. ICI-induced HPD is complex, and in some patients, the tumor size increases dramatically after checkpoint blockade. Therefore, it is imperative to explore the etiology and pathogenesis of HPD and develop predictive and detection methods to prevent the cessation of immunotherapy.

In clinical practice, the identification of HPD, disease progression and pseudo-progression is of great significance. At present, TGK and TGR are the main indicators for evaluating HPD, and rapid progression and TTF are two alternative indicators. To date, we have not yet determined the exact incidence of HPD because of the uncertainties in current testing methods and the relatively small number of patients examined. In the future, accurate diagnostic tools should be applied to evaluate tumor changes. As for predictive indicators, most existing conclusions are derived from retrospective studies that explored the specific clinical characteristics of HPD patients, but different types of studies may produce conflicting conclusions, and there is still a lack of accurate predictive indicators for HPD. To further understand HPD, more prospective, randomized and controlled studies are needed to verify whether HPD is an independent type of ICI response after treatment, identify predictive indicators and develop consistent criteria. This will allow us to efficiently predict the response of personalized treatment and develop personalized treatment plans.

In this review, we proposed that factors such as tumor microenvironments, radiation therapy, age, increased tumor burden, gut and tumor microbiomes, changes in immune cell subtypes, FcR polymorphisms, abnormal expression of tumor drivers or resistance-related genes and compensatory activation of other immune checkpoint pathways may be involved in the occurrence of HPD. Additionally, genomic analyses may help to elucidate the mechanisms of HPD and identify effective biomarkers to distinguish individuals at high-risk of HPD. Circulating tumor cell-free DNA may be used as a biomarker in the early monitoring and diagnosis of HPD. This will require large cohort studies to validate these potential detection methods.

In summary, HPD is a new phenomenon, and its underlying mechanism remains to be elucidated. In addition, the clinical relevance and predictive factors of HPD should be further investigated. HPD provides a challenge and an opportunity for the development of novel tumor biotherapies, and it will also encourage research to improve the effectiveness and safety of ICIs. It is important to study this issue in depth to protect cancer patients from the potentially harmful side effects of ICI therapies.
